# Maturation of Gut Microbiota and Circulating Regulatory T Cells and Development of IgE Sensitization in Early Life

**DOI:** 10.3389/fimmu.2019.02494

**Published:** 2019-10-23

**Authors:** Terhi Ruohtula, Marcus C. de Goffau, Janne K. Nieminen, Jarno Honkanen, Heli Siljander, Anu-Maaria Hämäläinen, Aleksandr Peet, Vallo Tillmann, Jorma Ilonen, Onni Niemelä, Gjalt W. Welling, Mikael Knip, Hermie J. Harmsen, Outi Vaarala

**Affiliations:** ^1^Clinicum, University of Helsinki, Helsinki, Finland; ^2^Department of Medical Microbiology, University Medical Center Groningen, University of Groningen, Groningen, Netherlands; ^3^Children's Hospital, Helsinki University Hospital, University of Helsinki, Helsinki, Finland; ^4^Department of Pediatrics, Jorvi Hospital, Helsinki University Hospital, Espoo, Finland; ^5^Immunogenetics Laboratory, Institute of Biomedicine, University of Turku, Turku, Finland; ^6^Department of Pediatrics, Tartu University Hospital, University of Tartu, Tartu, Estonia; ^7^Department of Laboratory Medicine and Medical Research Unit, Seinäjoki Central Hospital and University of Tampere, Seinäjoki, Finland; ^8^Research Programs Unit, Diabetes and Obesity, University of Helsinki, Helsinki, Finland; ^9^Folkhälsan Research Center, Helsinki, Finland; ^10^Tampere Center for Child Health Research, Tampere University Hospital, Tampere, Finland

**Keywords:** regulatory T-cells, bifidobacteria, gut microbiome, atopic diseases, IgE

## Abstract

Recent studies suggest that the cross-talk between the gut microbiota and human immune system during the first year of life is an important regulator of the later development of atopic diseases. We explored the changes in the gut microbiota, blood regulatory T cells, and atopic sensitization in a birth-cohort of Estonian and Finnish children followed from 3 to 36 months of age. We describe here an infant Treg phenotype characterized by high Treg frequency, the maturation of Treg population characterized by a decrease in their frequency accompanied with an increase in the highly activated Treg cells. These changes in Treg population associated first with the relative abundance of Bifidobacterium longum followed by increasing colonization with butyrate producing bacteria. High bifidobacterial abundance in the neonatal microbiota appeared to be protective, while colonization with *Bacteroides* and *E. coli* was associated with later risk of allergy. Estonian children with lower risk of IgE mediated allergic diseases than Finnish children showed an earlier maturation of the gut microbiota, detected as earlier switch to an increasing abundance of butyrate-producing bacteria, combined with an earlier maturation of Treg cell phenotype and total IgE production. The children with established allergic diseases by age 3 showed a decreased abundance of butyrate producing *Faecalibacterium*. These results suggest that as well as the maintenance of a bifidobacterial dominated gut microbiota is important during the first weeks of life, the overtake by butyrate producing bacteria seems to be a beneficial shift, which should not be postponed.

## Introduction

Our understanding of the cross-talk between the gut microbiota and human immune system during the time when allergic sensitization develops is limited, although accumulating evidence indicates that the gut microbiome plays a pivotal role in the regulation of allergic immune responses (1–8). The composition of the gut microbiome is determined by our microbial environment, dietary factors, and genetic background (9). The evidence of the mechanisms of the gut microbiome as a regulator of the immune response is largely based on studies in animal models showing e.g., that the gut microbiome directly or in-directly via metabolites is able to modulate the permeability of the intestinal epithelium (10–12), and the differentiation and function of effector and regulatory T cells (Treg cells) (13–21), which could further influence the development of immune-mediated diseases, including allergic, inflammatory, and autoimmune diseases. In prospective studies of children, indirect *in vitro* based evidence suggests that metabolites of the gut microbiome from the infants with high risk of atopic diseases could indeed modulate Treg cell phenotype (6). Arrieta et al. showed that reduced levels of fecal acetate and dysregulation of enterohepatic metabolites was accompanied with the gut microbiome changes predicting the risk of asthma in CHILD cohort (5). Thus, although the altered gut microbiota composition has been associated with allergic responses in children, the understanding of the mechanisms linking the gut microbiota and altered immune deviation is limited in humans.

To investigate the relationship between the development of the intestinal microbiota, circulating Treg cells, and IgE sensitization against environmental allergens, we obtained repeated blood and feces samples during the first 3 years of life from a cohort of infants living in Estonia or Finland, the neighboring countries with distinct differences in the standard of living^1^^,^^2^ and incidence of allergic diseases (e.g., 12 month prevalence of asthma 9.3 vs. 19.0 %) (22, 23). We found that the composition of the neonatal gut microbiota associated with later risk of allergic sensitization and allergic diseases. Our study further shows that the maturation of the circulating Treg cells included an increase in the highly activated Treg cells, which was associated with the relative abundance of *Bifidobacterium longum* and colonization with butyrate producing bacteria, and this maturation process of the gut microbiota and Treg cells was delayed in Finnish children with a higher risk of IgE mediated allergic sensitization and diseases in comparison to Estonian children.

## Materials and Methods

### Study Subjects

We studied a subgroup of Estonian and Finnish children participating in the DIABIMMUNE (Pathogenesis of type 1 diabetes: testing the hygiene hypothesis) study (Estonia; *n* = 85; 43/42 and Finland; *n* = 76; 42/34 male/female). These index cases carried HLA conferred genetic risk for type 1 diabetes and celiac disease as previously described (24) High-risk genotype (DRB1^*^03-DQA1^*^05-DQB1^*^02 and DRB1^*^0401/2/4/5-DQA1^*^03-DQB1^*^03:02 haplotypes) was found in 12, moderate-risk genotypes in 52 (either one of the high-risk haplotypes or, DRB1^*^04:01/2/5-DQA1^*^03-DQB1^*^03:02 with a neutral haplotype), slightly increased risk genotypes in 92 (either the DRB1^*^04:04-DQA1^*^03-DQB1^*^03:02 or DRB1^*^03-DQA1^*^05-DQB1^*^02 haplotype with a neutral haplotype), and neutral haplotypes in 5 children. Neutral haplotypes are all other except the protection associated haplotypes DQB1^*^02, 03:01 or 06:02, or any of the risk haplotypes. Of all the infants 74 Estonian and 71 Finnish children (38/36; 40/31 male/female) were born vaginally. The present study was conducted according to the guidelines of the Declaration of Helsinki, and was approved by the ethical committees of both study centers, and written informed consent was obtained from the children's parents. The number of study subjects and samples analyzed in the different assays are given in Supplementary Table 1.

### Flow Cytometry Analysis of Circulating Regulatory T-Cells

We analyzed the phenotype of peripheral blood regulatory T-cells in fresh heparinized blood samples obtained at the age of 3, 6, 12, 24, and 36 months (Supplementary Table 1). At least 1 × 10^6^ events were acquired from each sample on a FACSCalibur™ and analyzed with the FlowJo™ software. The samples were compensated post acquisition with FlowJo™ software. The monoclonal antibodies are shown in Supplementary Table 2. To assess the number of circulating CD4+CD25highFOXP3+ T cells in the samples, we gated first CD4+ cells, and then the CD25+CD127–/lo population. The expression of FOXP3 protein was analyzed in these cell populations. CD4+CD25highFOXP3+ expression was quantified as median fluorescence intensity (MFI) in arbitrary units (AU) after subtraction of the negative-control antibody intensity. Intensity values over the 97.5 percentile of the negative-control antibody were regarded as positive. Intensities were calibrated to a set of particles containing known amounts of fluorescein isothiocyanate.

### Gene Expression Based Phenotypic Analysis of Circulating Regulatory T-Cells

We performed RT-qPCR analysis of enriched circulating regulatory T-cells at 6, 18, and 36 month samples (Supplementary Table 1). The product information for the reagents used is shown in Supplementary Table 2. Enrichment of Treg cells was made according to the manufacturer's instructions using MACS CD25+CD49d– magnetic beads with the exception that the magnetic labeling of CD25+ regulatory T cells, which was titrated to 2.5 μl of CD25 MicroBeads II per 10^7^ total cells for improved cell purity. In addition, the second enrichment elution with the MS column was omitted as it resulted in too high yield loss, resulting in mean 87.7% purity of the enriched Treg cells. An additional wash with PBS was added before the pellets were frozen at −70°C in RLT-lysis buffer for subsequent RT-qPCR analysis. Total RNA from purified Tregs was isolated with the Qiagen RNeasy Plus Micro kit according to the manufacturer's instructions. cDNA was synthesized using the random hexamer priming of the High Capacity cDNA Reverse Transcription kit according to the manufacturer's instructions. Real time PCR was performed with TaqMan Fast master Mix and StepOne Plus instrument. TaqMan Gene Expression Assays were used for real time PCR amplification of FOXP3, TGF-beta1, Helios, GATA-3, CTLA-4, IL-10, and IFN-γ. The endogenous reference used was the gene ribosomal 18S. To analyze the relative amount of mRNA of the gene of interest a comparative ΔΔ cycle threshold (Ct) method was used. An in-house control sample (calibrator sample) was used to control inter-assay variation. The calibrator sample was prepared from the phytohaemagglutinin-stimulated human PBMC derived RNA.

### Total and Allergen-Specific IgE Analysis

Total IgE and allergen-specific IgE concentrations were analyzed from serum samples (Supplementary Table 1) at the age of 6, 18, and 36 months by using the ImmunoCAP fluoroenzyme immunoassay (Supplementary Table 2). IgE to egg, cow's milk, house dust mite, cat, timothy grass, and birch, as well as total IgE concentrations were analyzed at the age of 6 months. Peanut was added to the panel at the age of 18 months and dog at the age of 36 months. The children were classified into four groups based on their clinical allergy diagnosis and allergen-specific IgE values: (A) No signs of allergy (no clinical diagnosis, no specific IgE responses); (B) IgE-sensitization during the study period, up to 36 months of age (no clinical diagnosis, at least one specific IgE response); (C) sensitized, up to the time point analyzed (no clinical diagnosis, at least one specific IgE response); (D) clinical non-IgE allergy [clinical diagnosis, no specific IgE response (25)]; and (E) clinical IgE allergy (clinical diagnosis and a specific IgE response to the same allergen).

### Analysis of Microbiota

Total DNA was extracted from a 0.25 g fecal sample using the repeated bead beating method described in detail by Yu and Morrison (26), with a number of modifications. In brief, four 3 mm instead of 0.5 mm glass beads were added during the homogenization step. Bead beating was performed using a Precellys 24 at 5.5 beats per millisecond in three rounds of 1 min each with 30 s pauses at room temperature in between. The incubation temperature after the bead beating was raised from 70 to 95°C. Importantly, protein precipitation with 260 μl of ammonium acetate was carried out twice instead of only once. Elution of DNA from the purification columns was done twice. Columns from the QiaAmp Stool Kit were replaced by those from the QIAamp DNA Stool Mini Kit. The V3–V4 region of the 16S rRNA gene was amplified from the fecal DNA by polymerase chain reaction (PCR) using modified 341F and 806R primers with a 6 nucleotide barcode on the 806R primer. The sequence of the 341F primer and the 806R primer was aatgatacggcgaccaccgagatctacactctttccctacacgacgctcttccgatctNNNNCCTACGGGAGGCAGCAG & caagcagaagacggcatacgagat**CGTGAT**gtgactggagttcagacgtgtgctcttccgatctGGACTACHVGGGTWTCTAAT, respectively, where lowercase letters denote adapter sequences necessary for binding to the flow cell, underlined lowercase are binding sites for the Illumina sequencing primers, bold uppercase highlight the index sequences as reported by Bartram et al. (27) and regular uppercase are the V3–V4 region primers (341F on for the forward primers and 806R for the reverse primers). The inclusion of four maximally degenerated bases (“NNNN”) maximizes diversity during the first four bases of the run. Reaction conditions consisted of an initial 94°C for 3 min followed by 32 cycles of 94°C for 45 s, 50°C for 60 s, and 72°C for 90 s, and a final extension of 72°C for 10 min. An agarose gel confirmed the presence of product (band at ~465 base pairs) in successfully amplified samples. The remainder of the PCR product (~45 μl) of each sample was mixed thoroughly with 25 μl Agencourt AMPure XP magnetic beads and were incubated at room temperature for 5 min. Beads were subsequently separated from the solution by placing the tubes in a magnetic bead separator for 2 min. After discarding the cleared solution the beads were washed twice by resuspending the beads in 200 μl freshly prepared 80% ethanol, incubating the tubes for 30 s in the magnetic bead separator and subsequently discarding the cleared solution. The pellet was subsequently air dried for 15 min and resuspended in 52.5 μl 10 mM Tris HCl pH 8.5 buffer. Fifty microliter of the cleared up solution is subsequently transferred to a new tube. The DNA concentration of each sample was measured using a Qubit® 2.0 fluorometer and the remainder of the sample was stored at −20°C until library normalization. Library normalization was done the day before running samples on the MiSeq by making 2 nM dilutions of each sample. Samples were pooled together by combining 5 μl of each diluted sample. Ten microliter of the sample pool and 10 μl 0.2 M NaOH were subsequently combined and incubated for 5 min to denature the sample DNA. To this 980 μl of the HT1 buffer from the MiSeq 2 × 300 kit was subsequently added. A denatured diluted PhiX solution was made by combining 2 μl of a 10 nM PhiX library with 3 μl 10 mM Tris HCl pH 8.5 buffer with 0.1% Tween 20. These 5 μl were mixed with 5 μl 0.2 M NaOH and incubated for 5 min at room temperature. The resulting 10 μl were subsequently mixed with 990 μl HT1 buffer. One hundred and fifty microliter of the diluted sample pool is combined with 50 μl of the diluted PhiX solution and was further diluted by adding 800 μl HT1 buffer. Six hundred microliter of the prepared library was loaded into the sample-loading reservoir of the MiSeq 2 × 300 cartridge.

### Statistical Analysis

MiSeq sequencing pipeline and statistical analysis Software that was used to analyze the data received from Illumina paired-end sequencing, included PANDAseq (28), QIIME (29) and ARB (30). Paired-end reads that were shorter than 400 BP or longer than 500 BP were discarded by PANDAseq. Statistical analyses were performed on the family, genus and species level. QIIME identified sequences down to the Family and Genus level while ARB was used for most genera (31, 32), as is typically possible for most fecal bacteria using 16S (31, 32), to identify sequences down to the species level as earlier described by de Goffau et al. (33, 34) using the latest SILVA reference database (35). Importantly, species level resolution was achieved for species from the *Bifidobacterium* genus. Principal component analysis (PCA) was performed to find clusters of similar groups of samples or species. PCA is an ordination method based on multivariate statistical analysis that maps the samples into a reduced number of relevant dimensions of variability. The hierarchical clustering analysis was performed with the Hierarchical Clustering Explorer 3.5. The Simpson index was used as a measure of microbial diversity. Non-parametric tests were used, as microbial abundances are rarely normally distributed.

The Mann-Whitney *U*-test, Spearman correlation analysis (rs), the Wilcoxon or χ^2^ tests were used as indicated. The use ± indicates that a standard deviation is given. All tests were two-tailed and a *p* < 0.05 was considered to indicate statistical significance. All statistical analyzes were performed using IBM® SPSS® Statistics 20.0. For the analysis of Flow cytometry data Graph Pad Prism 5 software was used.

## Results

### Maturation Steps of Treg Cells During the First 3 Years of Life

**Figure 1:**
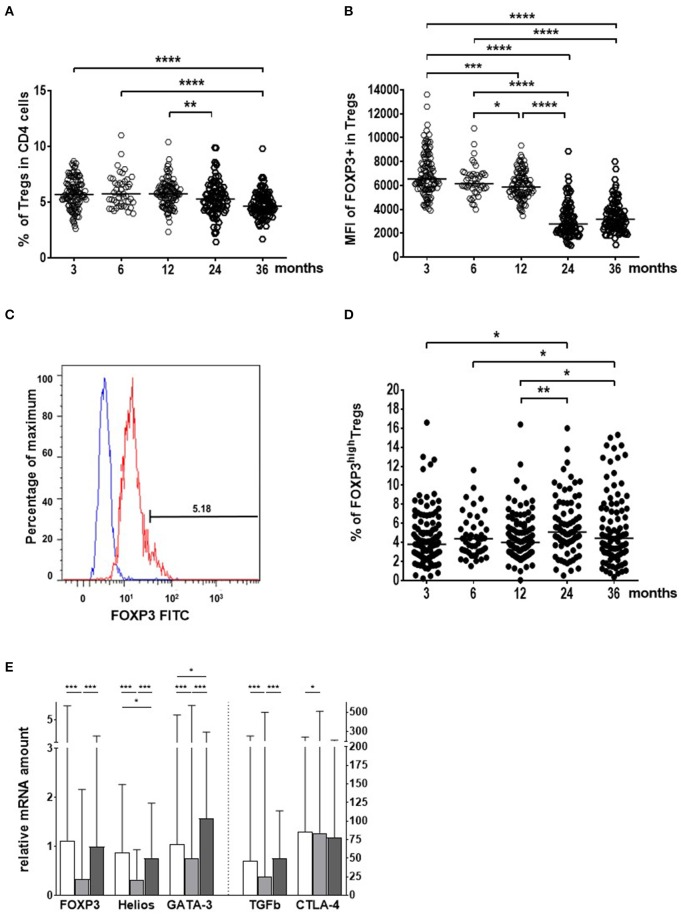
Age-related changes in the blood regulatory T cell (Treg) population from the age of 3 to 36 months in Estonian and Finnish children. **(A)** The frequency (%) of Treg cells in the CD4+ cell population. **(B)** FOXP3 expression as the median fluorescence intensities (MFI) in Treg cells. **(C)** The gating for the identification of TregFOXP3High cells. **(D)** The proportion (%) of TregFOXP3High cells in the Treg population. Sample sizes for figures **(A,B,D)** are *n* = 111 at 3 mo; *n* = 45 at 6 mo; *n* = 100 at 12 mo; *n* = 84 at 24 mo; and *n* = 92 at 36 months of age. Medians are shown as horizontal lines **(E)**. The expression of Treg activation markers (mRNA) in Treg cells in all children studied. The columns are showing the medians with ranges. White columns indicate 6 month, light gray columns 18 month, and dark gray columns 36 month samples. The vertical dotted line indicates the use of two different scales on the y-axis. The age of the children (months), are marked on the x-axes in **(A,B,D)** and the different activation markers in **(E)** (*n* = 33 at 6 mo; *n* = 96 at 18 mo; and *n* = 80 at 36 months of age). Wilcoxon test (two-sided) was used for comparisons. *P*-values in the figure are shown as ****p < 0.0001; ***p < 0.001; **p < 0.01 and *p < 0.05.

To investigate the early maturation of Treg cells we followed the characteristics of blood Treg cells at the ages of 3, 6, 12, 24, and 36 months in the Estonian and Finnish children. During the first year of life, the numbers of blood Tregs (CD4+CD127–/loCD25high) as well as their FOXP3 protein expression was high, a drop occurred in the relative numbers of Tregs and their FOXP3 expression after 12 months of age (Figures 1A,B). At the same time when the frequency of blood Treg cells decreased, the proportion of highly activated TregFOXP3high cells increased (Figures 1C,D), particularly in the Finnish children (Figure 1D). Similarly, the expression of FOXP3, Helios, TGF-beta1, CTLA-4, and GATA-3 transcripts in Tregs decreased from 6 to 18 months and then increased to 36 months (Figure 1E). Our results revealed a two-step maturation process in the circulating Treg cells: a decrease in the proportion of Treg cells followed by an increase in the highly activated Treg cells, which are reported to show enhanced suppressive activity (36–40).

### Bifidobacterial Composition at 3 Months Shapes Treg Cells

**Figure 2:**
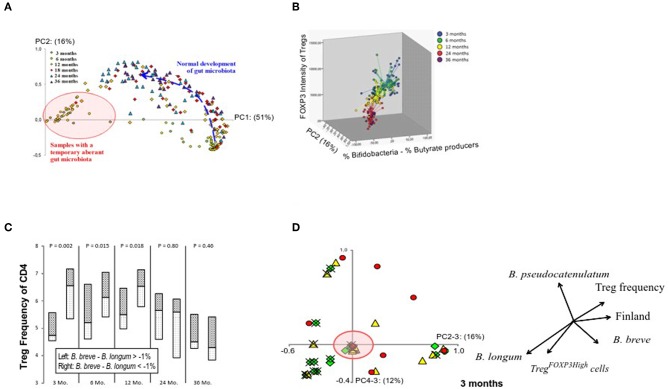
Relation between the maturation of the gut microbiota composition and Treg phenotype. **(A)** Principal component analysis on all samples on the genus level shows the age-related maturation of gut microbiome. Principal component 1 on the x-axis (from 0 to 1) accounts for 51% of the variation in the data while principal component 2 on the y-axis accounts for 16%. Samples at 3 and 6 months are either found in the lower right corner (normal bifidobacteria-dominated microbiota) or in the left (red circle) representing an aberrant microbiota largely devoid of bifidobacteria and instead replaced by a combination of *Escherichia coli, Bacteroides, Ruminococcus gnavus*, and/or clostridia (pink circle). The normal bifidobacteria-dominated microbiota (lower right corner) usually is the basis for future development (blue arrow) into a more complex adult-like configuration. **(B)** A graphical representation of FOXP3 expression levels in the whole Treg population in relation to the gut microbiota development. **(C)** A graphical representation of the influence of the bifidobacteria composition at 3 months of age on Treg numbers at 3, 6, 12, 24, and 36 months. Children with a *Bifidobacterium breve*—*Bifidobacterium longum* abundance lower than −1% at 3 months are depicted on the left of each column and those with scores higher than −1% are depicted on the right, with median as a horizontal line in the boxes. *P*-values (top) were not based on the binary division of 1% but on the actual *B. breve*—*B. longum* abundance per sample and their corresponding Treg-value using a Spearman rho's correlation test. The value of −1% was chosen to specifically showcase those children who have *B. longum* in a non-insignificant amount and have more of it than *B. breve*. **(D)** The number of Treg cells with high expression of FOXP3 shows association with PC4, which is described by the relative abundances of *B. longum* and *B. pseudocatenulatum* at the age of 3 months and accounts for 12% of the variation. The arrows in the image are a direct representation of the Spearman correlation coefficient of each of the indicated factors with the respective principal components. The green diamonds (♢) represent TregFOXP3High frequencies >4.5%; yellow triangles (▴) 3% < TregFOXP3High >4.5%; and red circles (•) TregFOXP3High frequencies <3%. Estonian children are marked with an (×). The red circle indicates samples, which represent an aberrant microbiota composition, and hardly contain bifidobacteria as also illustrated in **(A)**.

We hypothesized that gut colonization could drive the changes in the Treg cell population. In 16S rDNA sequencing of fecal samples, bifidobacteria comprised the most dominant microbial group at 3 and 6 months (Supplementary Figure 1). Principal component 1 (PC1, variance of 51%) differentiated the children with a normal bifidobacteria dominated infant microbiota and those with an aberrant microbiota at 3 months (Figure 2A). A shift toward microbiota dominated by butyrate producers occurred subsequently (Supplementary Figure 1), and this correlated with the changes in the FOXP3 intensity in Treg cells (Figure 2B).

At 3 months of age, PC2 (16%), which is described by the abundance of *Bifidobacterium breve* minus the abundance of *B. longum*, showed a positive correlation with the relative numbers of Treg cells (*p* = 0.006; rs = 0.40). Also, the linear combination of these bifidobacterial species (*B. breve*—*B. longum*) correlated with the numbers of Treg cells at 3 months (*p* = 0.002; rs = 0.44), and further later at 6 and 12 months (*p* = 0.015; rs = 0.33; and *p* = 0.018; rs = 0.35, respectively) suggesting that the initial bifidobacterial composition at 3 months has a long-term effect on Treg cell numbers (Figure 2C).

The proportion of highly activated TregFOXP3high cells at 3 months showed an association with PC4 (12%) (Figure 2D), which was almost totally defined by the abundance of *B. longum* minus the amount of *B. pseudocatenulatum* (*p* < 0.001; rs = 0.80), and consequently *B. longum* minus *B. pseudocatenulatum* associated with the proportion of TregFOXP3high cells (*p* = 0.007; rs = 0.40).

According to our results, bifidobacterial species, as suggested by earlier studies (21, 41–43), play an important role in shaping the Treg cell population, and the relative abundance of *B. longum* in the neonatal microbiota seems to be a promoter of Treg cell maturation, such as the subsequent decrease in the Treg numbers and the increase in the relative numbers of highly activated TregFOXP3high cells.

### Delayed Maturation of Microbiome and Treg Cells in Finnish Children

**Figure 3:**
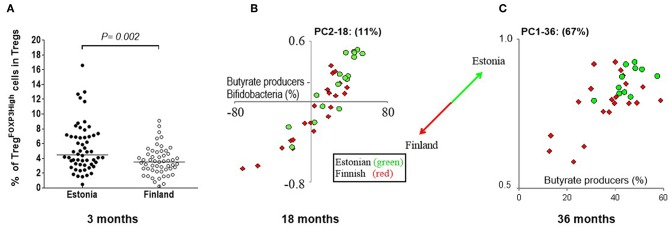
The differences in Treg cells at 3 months and gut microbiome between Estonian and Finnish children at 18 and 36 months of age. **(A)** The frequencies of TregFOXP3high cells in the Treg population is higher at 3 months in Estonian (*n* = 56) than Finnish children (*n* = 55). Median values are shown with a horizontal line. The *P*-value was calculated with Mann-Whitney U (two-sided). **(B)** PC2 at 18 months is mostly driven by the amount of butyrate produces minus the amount of bifidobacteria (X-axis), as can be seen by the strong positive correlation. Estonian children score higher on PC2 (18 month samples) than Finnish children, which indicates that Estonians have more butyrate producers at 18 months than the Finnish children, while the Finnish children have more bifidobacteria. At 36 months **(C)** another pattern is seen, this time described by PC1 (67%). Estonian children on average score higher on PC1 (Y-axis), which in turn is driven mostly by the abundance of butyrate producers in the 36 month samples (X-axis). Estonian children have on average more butyrate producers than then Finnish at 36 months. The circles (•) show Estonian and diamonds (♢) Finnish children in these principal component plots.

We had a unique opportunity to compare the development of Treg cells between Estonian and Finnish children. At 3 months, Estonian children had more highly activated TregFOXP3high cells in comparison to Finnish children (Figure 3A) in whom an increase in activated TregFOXP3high cells occurred after 3 months of age (Figure 1C). Furthermore, a positive correlation between FOXP3 and CTLA-4 transcripts, which reflect functional activity of Tregs, was seen in Estonian children earlier, at the age of 6 months (*p* = 0.008; rs = 0.59 in Estonian children and *p* = ns in Finnish children), while this kind of correlation between FOXP3 and CTLA-4 developed later in Finnish children and was seen at the age of 3 years (*p* < 0.001 for both at 36 months, rs = 0.58 and rs = 0.70 for Estonian and Finnish children, respectively). These findings suggest that the phenotype of blood Tregs remains longer immature during early life in Finnish children in comparison to Estonian children.

Also differences in the gut microbiota at 3 months of age was seen between Estonian and Finnish children, when the abundance of *B. breve* minus *B. longum* was higher in Finnish children (*p* < 0.001). At 18 months, PC2 (11%), which describes the abundance of well-established butyrate producers in relation to bifidobacteria, differentiates Estonian and Finnish children (Figure 3B). Estonian children had a lower bifidobacterial abundance than Finnish children (13% vs. 26%, *p* = 0.02), and a higher abundance of butyrate producing bacteria (40% vs. 29%, *p* = 0.01, Figure 3B) (for the butyrate producing bacteria see Supplementary Table 3). A drop in bifidobacteria took place only after 18 months in Finnish children. Despite of this, Estonian children scored higher at 36 months in PC1, which represents 67% of variation and primarily reflects the abundance of butyrate producers (Figure 3C). Consequently, a higher abundance of butyrate producers was found in Estonian children at 36 months (45% vs. 36%, *p* = 0.02) indicating a more mature microbiome composition. In addition, Finnish children showed a higher abundance of *Bacteroides* at both 12 and 18 months of age (9.7% vs. 2.4%, *p* = 0.003 and 6.6% vs. 1.6%, *p* = 0.008).

### Neonatal Gut Microbiota Predicts Allergic Response

**Figure 4:**
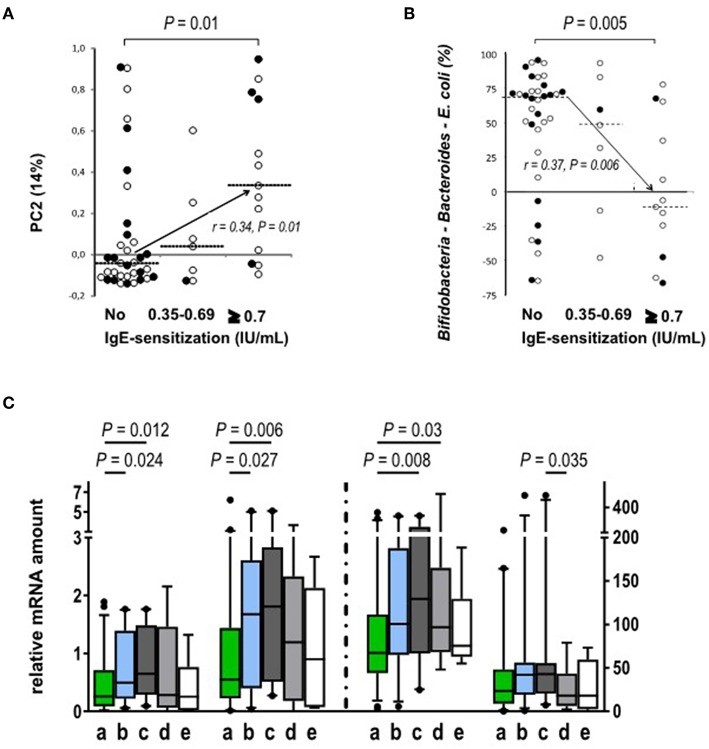
Relation between atopic sensitization and the gut microbiota composition and Treg phenotype. **(A)** PC2, as derived from the genus level at 3 months of age, correlates positively with atopic sensitization during the study period (*p* = 0.01, rs = 0.34; No sensitization *n* = 35; sensitization between 0.35 and 0.69 *n* = 7; sensitization ≥0.7 *n* = 13). **(B)** The abundances of *Bacteroides* and *E. coli*. are individually significantly associated with the development of atopic sensitization while bifidobacteria are protective. When both Gram negative groups are subtracted from the abundance of bifidobacteria the resulting association inversely mirrors the association of PC2 with atopic sensitization (*p* = 0.005; rs = 0.37; No sensitization *n* = 33; Allergen specific IgE level between 0.35 and 0.69 *n* = 7; Allergen specific IgE level ≥ 0.7 *n* = 12). Open circles show Finnish (°) and black circles show Estonian (•) infants. **(C)** The expression (mRNA) of activation markers FOXP3, GATA-3, and CTLA-4 is increased in blood derived Treg cell population at the age of 18 months in the children with IgE sensitization but without clinical allergy. The children were classified into four groups based on their clinical allergy diagnosis and allergen-specific IgE values: (a) No signs of allergy (no clinical diagnosis, no allergen-specific IgE responses, *n* = 46 for FOXP3 and TGFb-1 and *n* = 45 for GATA-3 and CTLA-4); (b) IgE-sensitization developed during the study period (no clinical allergy diagnosis, at least one allergen specific IgE response ≥0.35 IU/mL, *n* = 28); (c) IgE-sensitized at the time of follow-up point (no clinical allergy diagnosis, at least one allergen specific IgE response ≥0.35 IU/mL, *n* = 20 for all other and *n* = 21 for TGFb-1); (d) clinical non-IgE allergy (clinical diagnosis of allergy, no allergen specific IgE response 45, *n* = 16); and (e) clinical IgE allergy (clinical diagnosis of allergy and a specific IgE response to the allergen related to the symptoms, *n* = 6). The different activation markers are marked on the x-axes. The vertical dotted line indicates the use of two different scales on the y-axis. Medians are marked with a horizontal line. Mann-Whitney *U*-test (two-sided) was used for comparisons.

Next, we studied the association between the development of circulating allergen-specific IgE antibodies and their association with the composition of gut microbiota. We found that gut microbiota composition at 3 months of age, but not after that, showed an association with atopic sensitization, i.e., the development of allergen-specific IgE later in life. PC2 (genus level) shows association with atopic sensitization (Figure 4A) and it is nearly perfectly described by the abundance of bifidobacteria minus the abundances of *Bacteroides* and *E. coli* at 3 months of age (*p* < 0.001, rs = 0.96), which further shows an inverse association with atopic sensitization (Figure 4B) and represents a clear vector associated with the number of allergen-specific IgEs (*p* = 0.012), the age of the first allergen-specific IgE (*p* = 0.039) and also the diagnosis of allergies (*p* = 0.012). Furthermore, bifidobacteria and in particular the relative abundance of *B. longum* at 3 months showed an inverse association with atopic sensitization, the number of allergen-specific IgEs and the development of allergy (*p* = 0.023, *p* = 0.026, *p* = 0.022). As a conclusion, high abundance of *Bacteroides* and *E. coli* at the expense of the abundance of bifidobacteria in early infancy increased the risk of atopic sensitization and clinical allergy later in childhood.

At the age of 18 months, the expression of FOXP3, GATA-3, and CTLA-4 in Tregs was higher in the children with allergen-specific IgE sensitization without signs of clinical allergy when compared to the children who did not develop clinical allergies (Figure 4C).

**Figure 5:**
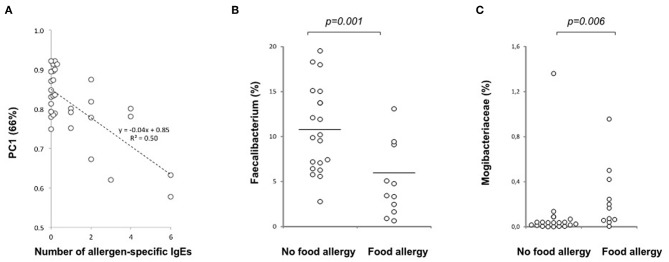
Relation of the gut microbiota composition and allergy at 36 months of age. **(A)** A negative correlation is seen between PC1 and the number of allergen-specific IgEs at 36 months of age (*p* < 0.001, *n* = 30), **(B)** Decreased abundance of the butyrate producer *Faecalibacterium prausnitzii* in children with food allergy at 36 months of age (*p* = 0.001; No food allergy *n* = 19, Food allergy *n* = 11). **(C)** Increased abundance of *Mogibacteriaceae* in children with food allergy at the age of 36 months (*p* = 0.006, *n* = 19 and 11, respectively). Medians are marked with a horizontal line.

At the end of the follow-up, atopic sensitization correlated (rs = 0.57; *P* < 0.001) with PC1 on the genus level, which accounts for 67% of the variation at 36 months of age, and describes the abundance of a trophic network of butyrate producers. PC1 is inversely correlated with the number of allergen-specific IgEs (rs = −0.50; *P* < 0.001) (Figure 5A), and atopy (rs = −0.57; *P* < 0.001), diagnosis of food allergies (rs = −0.58; *P* < 0.001) and inhalation allergies (rs = −0.39; *P* = 0.031).

In children with food allergy, *Faecalibacterium* (*F. prausnitzii*) (Figure 5B), which is directly correlated with PC1 and is one of the most important butyrate producing groups, was decreased while the abundance of *Mogibacteriaceae* (Figure 5C) being inversely correlated with PC1, was increased.

### Increased Total IgE Production in Estonian Children

Because total IgE levels are considered as a predictive marker for development of later IgE-sensitization and allergies, we analyzed the associations between total IgE levels, gut microbiota composition and characteristics of Tregs. Interestingly, we found that total IgE levels were higher in Estonian than Finnish children in the blood samples collected at 6 months (median 8.4, range 2–311 vs. 5.2 range 2–311293.5 kU/L; *p* = 0.02) and 36 months (62.4; range 2.41–2956 vs. 25.9; range 1.26–456 kU/L; *p* = 0.006, respectively). In Estonian children, total IgE levels at 6 months did not correlate with later IgE-sensitization or allergies as they did in Finnish children (*p* < 0.001, rs = 0.40).

As the dynamic changes in GATA-3 and FOXP3 expression in Treg cells have been shown to regulate the function of Treg cells and Th2 immunity (44, 45), we next asked whether GATA-3 mRNA expression in Treg cells is associated with the different kinetics seen in the maturation of IgE production. Indeed, in Estonian children, GATA-3 expression in Treg cells at 6 months correlated positively with total IgE levels at 18 months (*P* = 0.047, rs = 0.56) while in Finnish children GATA-3 expression level in Treg cells at 6 months showed an inverse correlation with total IgE levels at 6 (*P* = 0.001, rs = −0.77) and later at 18 months (*P* = 0.052, rs = −0.55). Similarly, Helios expression (46, 47) in Treg cells at 6 months correlated negatively with the total IgE production in Finnish children at 6 and 18 months of age (*P* = 0.002, rs = −0.75 and *P* = 0.021, rs = −0.63).

## Discussion

In the birth-cohort of Estonian and Finnish children followed from 3 to 36 months of age, we demonstrate that the composition of neonatal intestinal microbiota modulates the maturation of Treg population during the first year of life, and the risk of allergic diseases later in life. An earlier study in a US cohort identified a specific neonatal gut microbiota composition, which is characterized by a lower relative abundance of *Bifidobacterium, Akkermansia*, and *Faecalibacterium*, and a higher relative abundance of particular fungi, as a risk of atopy and asthma in a follow-up up to 4 years of age (6). That study also showed that fecal water extract from high-risk infants compromised Treg suppressive activity *in vitro* (6) suggesting that a defective function of Treg cells could be a factor contributing to the development of asthma. Those observations together with our findings support the view that neonatal microbiome is an important regulator of Treg cells and allergic sensitization. Our findings together with earlier studies emphasize the importance of the intestinal colonization during the first weeks or months of life as a key determinant for the risk of atopic deviation, and particularly the maintenance of a bifidobacterial dominated microbiome (2–7). Altogether, these studies suggest that the window of opportunity for the primary prevention of atopic diseases leading to asthma may be narrow, from birth up to 3 months of age.

In addition, we found that Finnish children, having higher risk of IgE mediated allergic diseases, show signs of delayed maturation of the gut microbiota and Treg cell phenotype when compared to Estonian children. Immature phenotype of the gut microbiota was found to be a risk factor for later allergic asthma in a Danish COPSAC cohort study (7). Accordingly, as well as the maintenance of a bifidobacterial dominated gut microbiota is important during the first weeks of life, the overtake by butyrate producing bacteria seems to be a beneficial shift, which should not be postponed. Butyrate is the main energy source for colonic epithelial cells (48), and it regulates the assembly of tight junctions and gut permeability (49). Thus, the switch to an increasing abundance of butyrate producing bacteria may facilitate the maturation of the gut barrier mechanisms during the early life (50). Also in children with already established allergic diseases, we found a decrease in butyrate producing *Faecalibacterium*, which is in agreement with the findings in the American Gut Project (51). Recent studies suggest the importance of butyrate in the regulation of allergic airway inflammation by limiting eosinophilic inflammation (52) and type 2 innate lymphoid-cell proliferation and function (53).

According to our findings, remarkable age-related changes occur in the phenotype of the circulating Treg population during the first years of life, and these changes are tightly linked to the maturation of the intestinal microbiota. The neonatal Treg phenotype is characterized by the high frequency of circulating Treg cells, which is in agreement with an earlier study reporting increased numbers of Tregs in infants compared to adults (54). An increase in Treg cells after birth is characterized by a high frequency of naïve Treg cells (39, 40). The high ratio of *B. breve* vs. *B. longum* in the gut microbiota at 3 months of age showed association with high Treg numbers during the whole first year of life emphasizing the long-term effects of the neonatal gut microbiome. The following step of Treg maturation, i.e., the emergence of a highly activated Treg cell phenotype with high expression of FOXP3, TGF-beta1 and Helios showed instead an association with the high relative abundance of *B. longum*. Indeed, bifidobacteria species have been demonstrated to affect the induction of FOXP3 Treg cells (21). *B. longum* can activate Treg cells in newborn and adult mice, whereas *B. breve* shows an effect on regulatory T cells only in newborn mice. Furthermore, supplementation with *B. longum* protected against airway inflammation in an animal model (21). Also in humans, feeding a *B. longum* strain induced FOXP3 expressing regulatory T cells (55). The beneficial effects of *B. longum* 35624 has been suggested to be mediated by surface associated exopolysaccharide (41). A recent study showed that administration of *B. longum* changes the microbiota composition and thus metabolic mediators (42). Importantly, it seems that the effects of bifidobacteria on Tregs are dependent on species and strains, which should be considered in the design of possible probiotic treatments as suggested by others as well (43).

Whereas, the abundance of *B. longum* was an important determinant of Treg cell maturation during early infancy, the maturation of Treg cells after the first year of life, i.e., increasing numbers of highly activated Treg cells, showed a strong association with the increasing abundance of butyrate producing bacteria, and thus increasing diversity of the intestinal microbiome, which is likely contributed by changes in the diet. Our findings linking the temporal changes in the gut microbiome with Treg cell maturation are supported by an earlier study showing that Treg maturation between 18 and 36 months of age includes dynamic changes in the homing receptor pattern from a4b7-integrin, a gut homing receptor, to CCR4, which attract T-cells to non-gastrointestinal tissues (40). Accordingly, the early maturation of Tregs in the infants seems to be driven by intestinal antigens, such as diet and microbiome derived antigens. The effects of gut microbiome may be local in the intestine, such as reported for the extrathymic effects of butyrate on Treg differentiation (13, 19). On the other hand, changes in the gut microbiota and metabolites during colonization could affect the maturation process of Treg cells in thymus, as it has been shown that changes in the intestinal microbiota induced by antibiotic treatment can affect the thymus microenvironment, namely the T cell receptor repertoire of thymic Treg cells (56).

We had an opportunity to compare Finnish and Estonian children who live in a different environment regarding the risk for allergy and standard of living. We observed several signs of delayed maturation of Treg cells in Finnish children in comparison the Estonian children, such as a lower frequency of the highly activated Treg cells at 3 months of age, and later development of the correlation between FOXP3 and CTLA-4 transcripts regulating immune suppression activity in the Treg cell population. Related differences in the gut microbiota between Estonian and Finnish children were evident, i.e., higher ratio of *B. longum* vs. *B. breve* at 3 months, and an earlier switch from bifidobacteria dominated microbiota to a microbiota with increasing numbers of butyrate producers in Estonian children.

Finally, despite having less IgE mediated allergies, Estonian children had higher total IgE levels than Finnish children indicating an earlier maturation of IgE production, which takes place during the first 5 to 6 years of life when the IgE levels reach adult levels (57). Differential maturation of IgE production between populations has been observed earlier indicating the importance of environmental factors (58). In Estonian children high total IgE did not associate with IgE sensitization and the risk of allergic diseases like in Finnish children suggesting differential regulation of IgE responses. In Estonian children GATA-3 expression in Tregs at 6 months of age was associated with total IgE production and correlated with the expression of CTLA-4, which is a functional mediator of immune suppression. Interestingly, the expression of FOXP3, CTLA-4, and GATA-3 in Treg cells were increased in children with IgE sensitization who did not develop clinical allergy, which suggests that a transcriptional signature supporting Treg suppression activity, i.e., FOXP3 and CTLA-4, could provide homeostasis despite of up-regulation of Th2 transcription factor GATA-3 in Treg cells (59).

We recognize that our study has limitations due to its observational nature, and due to the possible confounding factors, such as reported higher frequency of infections, and use of antibiotics and antipyretic drugs in Finnish children (60). On the other hand, it is among the first studies, in which the immunological maturation and changes in the composition of the gut microbiota are monitored in healthy children at the same time during the first years of life. Similar kind of temporal changes in the gut microbiota as seen in our cohort were described recently in TEDDY cohort (61, 62). Also several observations we made here, linking the immunological changes with the maturation of gut microbiota, are mechanistically supported by the earlier literature showing more direct evidence, which has been possible to generate in animal models. In this longitudinal follow-up study, we were able to reveal that delayed maturation of the gut microbiota, Treg cell population and total IgE production are characteristics of children living in an environment with high risk of allergic diseases, which encourages treatments promoting healthy maturation of gut microbiota aimed at the prevention of allergies.

## Data Availability Statement

The raw data supporting the conclusions of this manuscript will be made available by the authors, without undue reservation, to any qualified researcher.

## Ethics Statement

The studies involving human participants were reviewed and approved by The Hospital District of Helsinki and Uusimaa, Children's and Adolescents' Diseases and Psychiatry Ethical Committee, Tartu Ülikooli inimuuringute eetika komitee, and Ethics Review Committee (ERC) on Human Research of the University of Tartu. Written informed consent to participate in this study was provided by the participants' legal guardian/next of kin.

## Author Contributions

OV, TR, MG, HH, JN, and MK planned, performed, and analyzed experiments. TR and MG performed data analysis and statistical analyzes. JN and JH were involved in data discussion and supervision of study design experiments. A-MH, AP, VT, ON, and HS collected and provided patient and/or control material and clinical characterization. JN, JH, TR, and MG planned experiments. JI, GW, and HH provided scientific and experimental input. MK was involved in study design, data discussion, and supervision of experiments. OV designed experiments, analyzed data, and supervised the study. OV, TR, and MG wrote the manuscript. All authors critically reviewed and approved the manuscript.

### Conflict of Interest

The authors declare that the research was conducted in the absence of any commercial or financial relationships that could be construed as a potential conflict of interest.

## Footnotes

^1^http://apps.who.int/nha/database

^2^http://wdi.worldbank.org
