# Supplementary material for: Maturation of Gut Microbiota and Circulating Regulatory T Cells and Development of IgE Sensitization in Early Life
Source: Front Immunol. 2019 Oct 23;10:2494. doi: 10.3389/fimmu.2019.02494 (PMC6842923; doi:10.3389/fimmu.2019.02494)
Supplement: Supplementary file 1 [file Data_Sheet_1.pdf]

Supplementary Material

1 Supplementary Tables

Supplementary Table 1. Number of study subjects and of samples and gender distribution.

Supplementary Table 2. Product information on the reagents used in the study.

Supplementary Table 3. Overview of butyrate producers.

2 Supplementary Figures

Supplementary Figure 1. Complete linkage clustering, in combination with a microbial profile at the genus level, in children's fecal samples from 3 months to 36 months of age.

1. Supplementary Tables

Supplementary Table 1. Number of study subjects and of samples and gender distribution.

Samples, N-values	Country	Treg, Flow cytometry		Purified Treg cells, mRNA analysis		Microbiota, Feces		Aberrant microbiota	Total IgE	
		**Total**	**Male**	**Total**	**Male**	**Total**	**Male**	**Male**	**Total**	**Male**
**Index cases**	Estonia	85	43	65	34	18 [7]*	5	0	70	33
	Finland	76	42	56	31	29 [11]*	13	6	72	40
**All Samples**	Estonia	199	104	108	59	96 [15]*	28	0	178	96
	Finland	233	123	111	63	147 [18]*	63	10	197	105
**Sample age:**										
**3 months**	Estonia	56	29			18	5	0		
	Finland	55	28			29	13	5		
**6 months**	Estonia	18	5			18	5	0	70	33
[N_{total} = 37 / 41 (E/F)]	Finland	27	12			29	13	5	72	40
Additional time points	Estonia					5**		0**		
	Finland					9**		4**		
Separate set of samples	Estonia			19	9					
	Finland			14	11					
**12 months**	Estonia	43	22			17	5			
	Finland	57	32			27	12			
**18 months**	Estonia			48	26	17	5		49	31
	Finland			48	24	22	8		62	30
**24 months**	Estonia	40	23			15	5			
	Finland	44	23			21	8			

**36 months**	Estonia	42	25	31	24	11	3	58	32
	Finland	50	28	49	28	20	10	62	34

* Aberrant microbiota N-values in brackets, included in the total.

** Additional feces samples from two and four month samples from the same infants with aberrant microbiota.

Supplementary Table 2. Product information on the reagents used in the study.

Product	clone	Product code	Supplier
perCP-anti-human-CD4	SK3	345770	BD Biosciences, Franklin Lakes, NJ, USA
APC-anti-human-CD25	M-A251	555434	BD Biosciences, Franklin Lakes, NJ, USA
PE-anti-human-CD127	HIL-7R-M21	557938	BD Biosciences, Franklin Lakes, NJ, USA
Alexa-488-anti-human-FOXP3	206D	320111	BioLegend, San Diego, CA, USA
Alexa488-Mouse-IgG1-isotype		400133	BioLegend, San Diego, CA, USA
SPHERO^{TM} Easy Calibration Fluorescent Particles, FITC		ECFP-F1-5K	Spherotech, Libertyville, IL, USA
SPHERO^{TM} Easy Calibration Fluorescent Particles, PE		ECFP-F2-5K	Spherotech, Libertyville, IL, USA
SPHERO^{TM} Easy Calibration Fluorescent Particles, APC		ACP30-5K	Spherotech, Libertyville, IL, USA
GE Healthcare's Ficoll-Paque density gradient		17-1440-03	Amersham Biosciences, Uppsala, Sweden
FACS Lysing Solution		349202	BD Biosciences, San Jose, CA
MACS $CD25^+CD49d^-$ Treg isolation kit		130-094-551	Miltenyi Biotec, Auburn, CA, USA
RLT-lysis buffer		79216	Qiagen, Hilden, Germany

Qiagen RNeasy Plus Micro kit	74034	Qiagen, Hilden, Germany
High Capacity cDNA Reverse Transcription kit	43-688-14	Applied Biosystems, Foster City, CA
TaqMan Fast master Mix	4367846	Applied Biosystems, Foster City, CA
CTLA-4 probe	Hs 00175480_ml	Applied Biosystems, Foster City, CA
FOXP3 probe	Hs00203958_m1	Applied Biosystems, Foster City, CA
GATA-3 probe	Hs00231122_m1	Applied Biosystems, Foster City, CA
Helios probe	Hs00212361_m1	Applied Biosystems, Foster City, CA
IFN-□□probe	Hs 00989291_ml	Applied Biosystems, Foster City, CA
IL-10 probe	Hs00155485_m	Applied Biosystems, Foster City, CA
TGF-beta1 probe	Hs 00171257_ml	Applied Biosystems, Foster City, CA
gene ribosomal 18S probe	Hs99999901_s1	Applied Biosystems, Foster City, CA
ImmunoCAP fluoroenzyme immunoassay	http://www.phadia.com/en	Phadia Diagnostics, Uppsala, Sweden
FACSCalibur™		BD Biosciences
FlowJo™ software		Tree Star, Ashland, OR, USA
StepOne Plus instrument		Applied Biosystems, Foster City, CA
TaqMan Gene Expression Assays		Applied Biosystems, Foster City, CA
Precellys 24		Bertin Technologies, Montigny le Bretonneux, France
QIAamp DNA Stool Mini Kit		Qiagen, Hilden, Germany
modified 341F primer		Illumina, Eindhoven, The Netherlands
modified 806R primer		Illumina, Eindhoven, The Netherlands
Agencourt AMPure XP magnetic beads		Beckman Coulter, Inc. Brea, CA
Qubit® 2.0 fluorometer		www.invitrogen.com/qubit

MiSeq Reagent Kit	Illumina, Eindhoven, The Netherlands
Graph Pad Prism 5 software	La Jolla, CA, USA

Supplementary Table 3. Overview of butyrate producers

Bacterial group	Reference
**Faecalibacterium genus**	
Faecalibacterium prausnitzii	[63]
**Roseburia-like genus**	
Eubacterium rectale	[64,65]
Roseburia intestinalis	[66]
Roseburia faecis	[67]
Roseburia hominis	[67]
**Coprococcus-like genus**	

Eubacterium hallii	[68]
Coprococcus eutactus	[68]
Coprococcus comes	[68]
Coprococcus catus	[68]
**Various Eubacteria**	
Eubacterium eligens	[68,69]
Eubacterium desmolans	[69]
Eubacterium ventriosum	[65,69]
Eubacterium ramulus	[65,69]
Eubacterium biforme	[65]

63. Duncan SH, Hold GL, Harmsen HJ, Stewart CS, Flint HJ. Growth requirements and fermentation products of Fusobacterium prausnitzii, and a proposal to reclassify it as Faecalibacterium prausnitzii gen. nov., comb. nov. Int J Syst Evol Microbiol. 2002;52(Pt 6):2141-6.

64. Duncan SH, Flint HJ. Proposal of a neotype strain (A1-86) for Eubacterium rectale. Request for an opinion. Int J Syst Evol Microbiol. 2008;58(Pt 7):1735-6.

65. Schwiertz A, Lehmann U, Jacobasch G, Blaut M. Influence of resistant starch on the SCFA production and cell counts of butyrate-producing Eubacterium spp. in the human intestine. J Appl Microbiol. 2002;93(1):157-62.

66. Duncan SH, Hold GL, Barcenilla A, Stewart CS, Flint HJ. Roseburia intestinalis sp. nov., a novel saccharolytic, butyrate-producing bacterium from human faeces. Int J Syst Evol Microbiol. 2002;52(Pt 5):1615-20.

67. Duncan SH, Aminov RI, Scott KP, Louis P, Stanton TB, Flint HJ. Proposal of Roseburia faecis sp. nov., Roseburia hominis sp. nov. and Roseburia inulinivorans sp. nov., based on isolates from human faeces. Int J Syst Evol Microbiol. 2006;56(Pt 10):2437-41.

68. Holdeman LV MW. New Genus, Coprococcus, Twelve New Species, and Emended Descriptions of Four Previously Described Species of Bacteria from Human Feces. Int J Syst Evol Microbiol. 1974;Apr;24((Pt 2)):260-77.

69. Barcenilla A, Pryde SE, Martin JC, Duncan SH, Stewart CS, Henderson C, et al. Phylogenetic relationships of butyrate-producing bacteria from the human gut. Appl Environ Microbiol. 2000;66(4):1654-61.

2. Supplementary Figures

3.

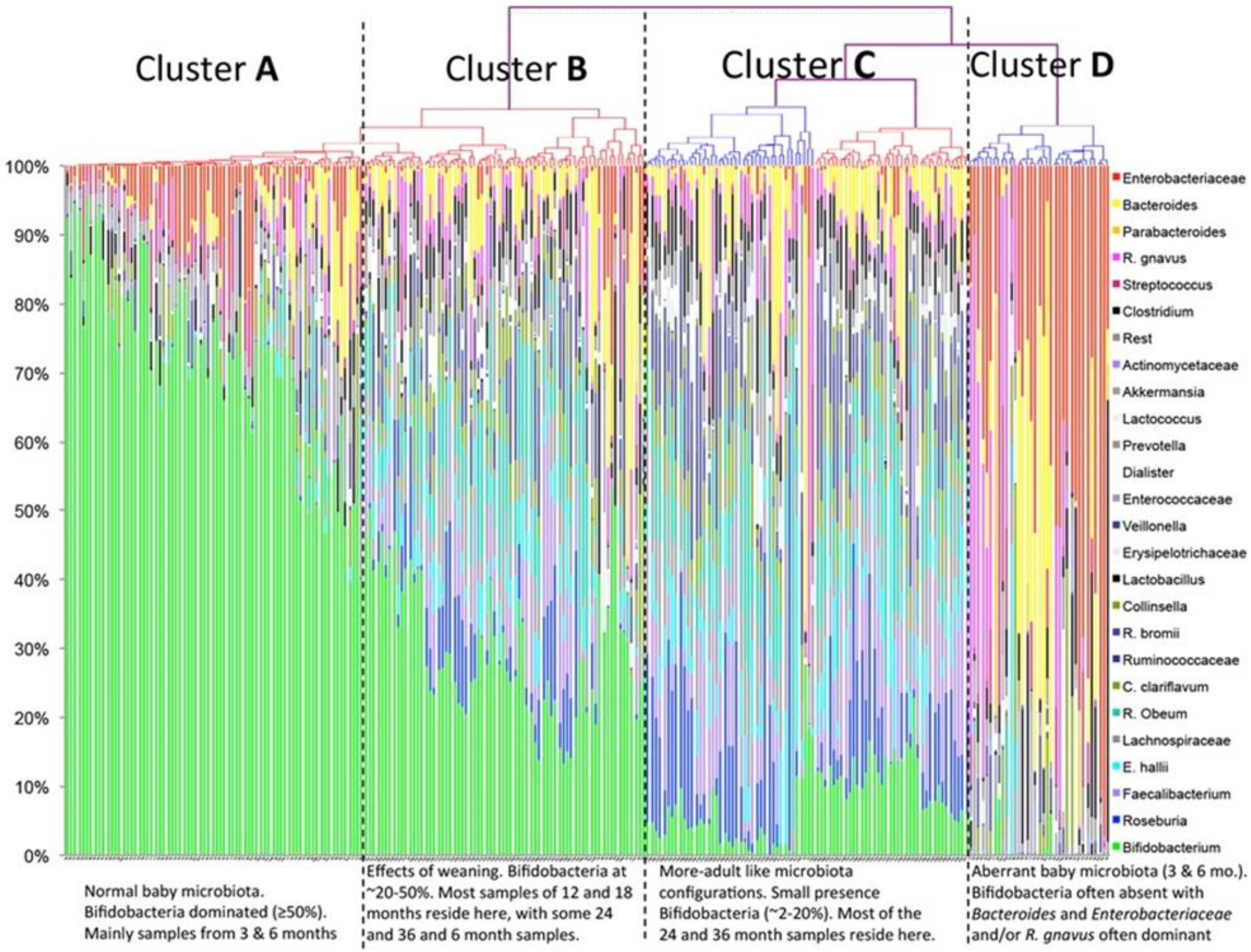
Cluster A
Cluster B
Cluster C
Cluster D
100%
90%
80%
70%
60%
50%
40%
30%
20%
10%
0%
Enterobacteriaceae
Bacteroides
Parabacteroides
R. gnavus
Streptococcus
Clostridium
Rest
Actinomycetaceae
Akkermansia
Lactococcus
Prevotella
Dialister
Enterococcaceae
Veillonella
Erysipelotrichaceae
Lactobacillus
Collinsella
R. bromii
Ruminococcaceae
C. clariflavum
R. Obeum
Lachnospiraceae
E. hallii
Faecalibacterium
Roseburia
Bifidobacterium
Normal baby microbiota. Bifidobacteria dominated (≥50%). Mainly samples from 3 & 6 months
Effects of weaning. Bifidobacteria at ~20-50%. Most samples of 12 and 18 months reside here, with some 24 and 36 and 6 month samples.
More-adult like microbiota configurations. Small presence Bifidobacteria (~2-20%). Most of the 24 and 36 month samples reside here.
Aberrant baby microbiota (3 & 6 mo.). Bifidobacteria often absent with Bacteroides and Enterobacteriaceae and/or R. gnavus often dominant

Supplementary Figure 1. Complete linkage clustering, in combination with a microbial profile at the genus level, in children's fecal samples from 3 months to 36 months of age. Cluster A. Bifidobacteria dominate (≥50%). Cluster B. Bifidobacteria ~20-50%. Cluster C. Bifidobacteria ~2-20%, an adult-like configuration. Cluster D. Aberrant microbiota consists mostly of samples taken at 3 and 6 months: Bifidobacteria are largely absent and a combination of Bacteroides, Escherichia coli and/or Ruminococcus gnavus is dominant.
